# Supplementary material for: Translation, cross-cultural adaptation, and psychometric evaluation of the Persian (Farsi) version of the QoLAF (quality of life in patients with anal fistula) questionnaire
Source: PLoS One. 2023 Apr 7;18(4):e0277170. doi: 10.1371/journal.pone.0277170 (PMC10081801; doi:10.1371/journal.pone.0277170)
Supplement: S1 File — (PDF) [file pone.0277170.s001.pdf]

Quality of Life in patients with Anal Fistula (QoLAF) Questionnaire (English* – Persian/Farsi**)

پرسشنامه کیفیت زندگی بیماران مبتلا به فیستول آنال (QoLAF) (انگلیسی* ، فارسی**)

Question in English Language *<br>سوال به زبان انگلیسی*	Question in Persian/Farsi Language **<br>سوال به زبان فارسی**	Question Number<br>شماره سوال
How often do you experience discharge (suppuration) from the fistula?	هر چند وقت يك بار از فيستول، ترشح (چرکي) داريد؟	**1**
How much discharge (suppuration) from the fistula do you experience?	چه مقدار ترشح (چرکي) از فيستول داريد؟	**2**
How often do you experience uncontrollable flatulence (farting) since having the fistula?	از زماني كه فيستول داريد، هر چند وقت يك بار خروج بي اختيار گاز از مقعد داريد؟	**3**
How often do you experience unintentional loss of stools since having the fistula?	از زماني كه فيستول داريد، هر چند وقت يك بار خروج بي اختيار مدفوع داريد؟	**4**
What is the amount of unintentional stool loss that you usually experience since having the fistula?	از زماني كه فيستول داريد، چه مقدار دفع مدفوع بي اختيار داريد؟	**5**
How often do you experience pain in the anal area as a consequence of the fistula?	هر چند وقت يك بار درد در ناحيه مقعد ناشي از فيستول داريد؟	**6**
What is the intensity of the pain that you experience as a consequence of the anal fistula?	شدت درد ناشي از فيستول مقعد چقدر است؟	**7**
Since suffering the symptoms of the anal fistula, how would you say your health is?	از زماني كه از علايم فيستول مقعد رنج مي بريد، سلامت خود را چگونه ارزيابي مي كنيد؟	**8**
How much does the anal fistula affect your physical health? (eg, energy and activity levels, sleeping pattern, general wellbeing…)	فيستول مقعد چه مقدار سلامتي جسمي شما را تحت تاثير قرار مي دهد؟ (مثل انرژي، ميزان فعاليت، الگوي خواب، تندرستي و ...)	**9**
How much does the anal fistula affect your psychological health? (eg, your body image, self-esteem, state of mind, ability to focus on a particular task…)	فيستول مقعد چه مقدار سلامتي رواني شما را تحت تاثير قرار مي دهد؟ (مثل تصوير ذهني از خود، اعتماد به نفس، وضعيت ذهني، توانايي تمركز روي يك كار خاص و ...)	**10**
How much does the anal fistula affect your independence level? (eg, mobility, ability to work, daily activities…)	فيستول مقعد چه مقدار سطح استقلال شما را تحت تاثير قرار مي دهد؟ (مثل تحرك، توانايي كار كردن، فعاليت هاي روزانه و ...)	**11**
How much does the anal fistula affect your social relationships and interactions with others? (eg, your relationships with friends, family, partner…)	فيستول مقعد چه مقدار ارتباطات اجتماعي شما و تعامل با ديگران را تحت تاثير قرار مي دهد؟ (مثل روابط با دوستان، خانواده ، شريك زندگي و...)	**12**
How much does the anal fistula affect your sexual relationships?	فيستول مقعد چه مقدار روابط جنسي شما راتحت تاثير قرار مي دهد؟	**13**
How much does the anal fistula affect other aspects of your life? (eg, your freedom, your economic income, your free time…)	فيستول مقعد چه مقدار ديگر جنبه هاي زندگي شما را تحت تاثير قرار مي دهد؟ (مثل آزادي، ميزان درآمد اقتصادي ، وقت آزاد و ...)	**14**

* **English Language:** Ferrer-Márquez M, Espínola-Cortés N, Reina-Duarte A, Granero-Molina J, Fernández-Sola C, Hernández-Padilla JM. Design and Psychometric Evaluation of the Quality of Life in Patients With Anal Fistula Questionnaire. Dis Colon Rectum. 2017 Oct;60(10):1083-1091. doi: 10.1097/DCR.0000000000000877. PMID: 28891853.

** **Persian/Farsi Language:** Keramati MR, et l.
