# Supplementary material for: Translation, cross-cultural adaptation, and psychometric evaluation of the Persian (Farsi) version of the QoLAF (quality of life in patients with anal fistula) questionnaire
Source: PLoS One. 2023 Apr 7;18(4):e0277170. doi: 10.1371/journal.pone.0277170 (PMC10081801; doi:10.1371/journal.pone.0277170)
Supplement: Attachment — Submitted filename: PLOS ONE REVIEW.docx [file pone.0277170.s005.docx]

**Reviewer Recommendation and Comments for Manuscript Number PONE-D-22-15392R1**

COMMENT:

The paper has been successfully reviewed the second time and I recommend that it is ACCEPTED for publication in this journal.

Thank you for given the opportunity to review this manuscript the second time.

Dr. Chinedu Godwin Uzomba
