# Supplementary material for: Translation, cross-cultural adaptation, and psychometric evaluation of the Persian (Farsi) version of the QoLAF (quality of life in patients with anal fistula) questionnaire
Source: PLoS One. 2023 Apr 7;18(4):e0277170. doi: 10.1371/journal.pone.0277170 (PMC10081801; doi:10.1371/journal.pone.0277170)
Supplement: Attachment — Submitted filename: REVIEWER COMMENT ON CROSS-CULTURAL ADAPTATION.docx [file pone.0277170.s003.docx]

Dear Authors,

I want to appreciate you for drafting this manuscript. It was well written and needed for the population.

However, there is a need for adjustments to the manuscript's structure. Firstly, I will suggest the titled be changed to “Translation, Cross-cultural Adaptation, and Psychometric evaluation of the Persian (Farsi) version of the QoLAF (Quality of Life in patients with Anal Fistula) questionnaire. Secondly, the following should be noted in the abstract:

1. The study did not capture which specific translation process used. It should be mentioned if it was based on the WHO translation process or any other that was specifically followed. The assessment of the manuscript would be based on which specific guideline that was used.
2. The results section should briefly capture the translation, what was found during the cross-cultural adaptation (CCA) and major psychometrics like validity and reliability.
3. There are too many unnecessary results in the abstract.

Finally, the authors should be able to distinguish between the three parameters of cross-cultural adaptation of a tool and present their results separately; 1. Translation 2. Adaptation 3. Psychometric property testing.

A clear distinction should be made between translation, adaptation and cross-cultural

validation. The translation is the single process of producing a document from a source version

in the target language. Adaptation refers to the process of considering any differences

between the source and the target culture to maintain equivalence in meaning. This

adaptation is referred to CCA. The cross-cultural validation of a questionnaire is a different process from the CCA. Cross-cultural validation aims to ensure that the new questionnaire functions as intended, has the same properties as the original, and functions similarly.

Kindly note that no results of the adaptation were presented in the manuscript.

A clear distinction should be made between translation, adaptation and cross-cultural

validation. The translation is the single process of producing a document from a source version

in the target language. Adaptation refers to the process of considering any differences

between the source and the target culture to maintain equivalence in meaning. This

adaptation is referred to CCA. The cross-cultural validation of a questionnaire is different

process from the CCA. Cross-cultural validation aims to ensure that the new questionnaire

functions as intended and has the same properties as the original and functions in the same

way.
